# Mesenteric lymph node transcriptome profiles in BALB/c mice sensitized to three common food allergens

**DOI:** 10.1186/1471-2164-12-12

**Published:** 2011-01-06

**Authors:** Mainul Husain, Herman J Boermans, Niel A Karrow

**Affiliations:** 1 Department of Animal & Poultry Science, University of Guelph, Guelph, Ontario, Canada N1G 2W1; 2 Department of Biomedical Sciences, Ontario Veterinary College, University of Guelph, Guelph, Ontario, Canada N1G 2W1; 3 Department of Pathology & Molecular Medicine, McMaster University, 1280 Main Street West, Hamilton, Ontario Canada L8S 4L8

## Abstract

**Background:**

Food allergy is a serious health concern among infants and young children. Although immunological mechanism of food allergy is well documented, the molecular mechanism(s) involved in food allergen sensitization have not been well characterized. Therefore, the present study analyzed the mesenteric lymph node (MLN) transcriptome profiles of BALB/c mice in response to three common food allergens.

**Results:**

Microarray analysis identified a total of 1361, 533 and 488 differentially expressed genes in response to β-lactoglobulin (BLG) from cow's milk, ovalbumin (OVA) from hen's egg white and peanut agglutinin (PNA) sensitizations, respectively (p < 0.05). A total of 150 genes were commonly expressed in all antigen sensitized groups. The expression of seven representative genes from microarray experiment was validated by real-time RT-PCR. All allergens induced significant ear swelling and serum IgG1 concentrations, whereas IgE concentrations were increased in BLG- and PNA-treated mice (p < 0.05). Treatment with OVA and PNA significantly induced plasma histamine concentrations (p < 0.05). The PCA demonstrated the presence of allergen-specific IgE in the serum of previously sensitized and challenged mice.

**Conclusions:**

Immunological profiles indicate that the allergen dosages used are sufficient to sensitize the BALB/c mice and to conduct transcriptome profiling. Microarray studies identified several differentially expressed genes in the sensitization phase of the food allergy. These findings will help to better understand the underlying molecular mechanism(s) of food allergen sensitizations and may be useful in identifying the potential biomarkers of food allergy.

## Background

Food allergy is serious health concern, affecting approximately 1-2% adults and 6-8% children [1]. Allergies to egg white and cow's milk are most common among children, whereas the most severe form of the food allergy is induced by peanuts [2]. Food allergy is typically considered an IgE-mediated type I hypersensitivity reaction [3]. Although increased production of IgE and Th2 cytokines (IL-4, IL-5 and IL-13) are considered common markers of food allergy [4], absence of IgE or increased levels of Th1 cytokines (IL-2, IFN-γ and TNF-α) have also been observed in several instances of food allergy [5,6], mainly in response to cow's milk [7,8]. Several animal studies have demonstrated that the involvement of the Th2 signaling pathway is too simplistic to explain the mechanism(s) of food allergies, since combined involvement of Th1 and Th2 signaling pathways have also been demonstrated [6,9]. Such uncertainty over the Th1/Th2 paradigm emphasizes the need for further research to unravel the molecular mechanism(s) of food allergy.

Although a genetic basis for IgE-mediated conditions such as asthma has been well documented, [10,11], little is known about the specific genes involved in the pathogenesis of food allergy. A study by de Jonge *et al. *investigated the mesenteric lymph node (MLN) gene expression profiles of Brown-Norway (BN) rats that were challenged with peanut extract, and they identified 64 differentially expressed genes [12]; the MLNs were ideal tissue for studying the mechanisms of food allergy because they provide a unique cellular and cytokine environment that is conducive to efficient antigen trapping and presentation to T-cells, the activation and proliferation of antigen-specific lymphocytes, and memory cell development [13,14]. In another study Cardoso *et al*. reported significant up-regulation of the Gata-3, IL-4, TNF-α, and IL-13 genes in the intestine of C57BL/6 mice sensitized with peanut extract and challenged with peanut seeds [15]. Although these differentially expressed genes may be potential risk factor candidates for peanut allergy, the induction of these genes may not necessarily be involved in the immune response to other food allergens. Recent studies indicate that Foxp3+ T regulatory cells may play a protective role in food allergy, and Foxp3 is considered an immunophenotype marker of T regulatory cells. In a recent report Krogulska *et al*. [16] suggested a possible protective role of Foxp3 and IL-10 in food allergy, where the expression of these genes was up-regulated in children who were developing tolerance to allergenic foods. Another recent report however, contradict to such tolerogenic effects of Foxp3 in food allergy, where Foxp3 along with Nfat-C2, IL-16 and Gata-3 genes were reported to up-regulated in children with persisting cow's milk allergy [17]. Such conflicting reports make it difficult to identify reliable biomarkers of food allergy. Therefore, it is necessary to continue further research to identify other biomarkers of food allergy.

A number of different animal models have been used to study food allergy [18-21]. Mice, however, are the most widely used model, and this is in part because immunological and molecular tools and reagents for this species are readily available. Also, their short breeding cycle and smaller size make them a cost effective model for conducting allergy research [22]. Of the different murine strains that are available, the BALB/c strain has been typically preferred over other strains because of its high IgE response that is characteristic of an atopic phenotype [20].

A suitable route of exposure is critical for studying the allergenicity of food proteins. Sensitization by intra-gastric (i.g.) gavage has been used by several groups [23,24], although there is a potential risk of developing oral tolerance via this route [25]. Adjuvants, such as cholera toxin, have also been used during oral sensitization, however, eliciting an immune response via this approach may not necessarily mimic the allergic response in humans [26], and may lead to misinterpretation of the data due to hyperstimulation of the immune system [27]. In order to avoid the development of tolerance and use of adjuvants, systemic exposure by intraperitoneal (i.p.) injection has been used by many groups, and this has been reported to be reliable and effective method for inducing sensitization [20,22].

In the following study, the transcriptome profile of MLN from BALB/c mice was assessed using cDNA microarrays to identify genes that are differentially expressed in response to i.p. sensitization with three common food allergens of varying potency; cow's milk β-lactoglobulin (BLG) < hen's egg ovalbumin (OVA) < peanut agglutinin (PNA) [20,28-30]. The sensitization concentrations of the antigens used in this study were shown to be sufficient to elicit type I allergic reactions during a subsequent challenge. The results presented here in this study may help to establish a method in future to identify the potential allergenicity of other food proteins. To the best of our knowledge, this study is the first to analyze the MLN transcriptome profile of BALB/c mice in response to BLG, OVA and PNA sensitization.

## Results

### Differential gene expression profiling by cDNA microarray analysis

**Figure 1:**
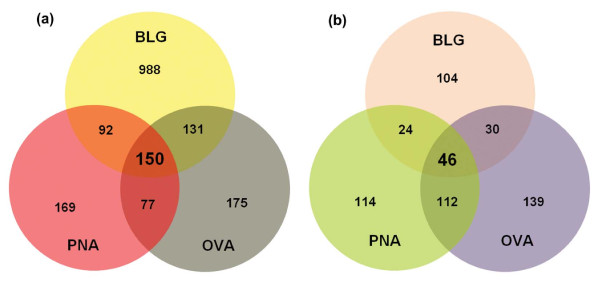
**Common and unique differentially expressed genes, and gene ontology biological processes**. (a) Genes significantly differentially expressed (p ≤ 0.05) in response to BLG, OVA or PNA sensitization. A total of 150 overlapping genes were commonly expressed among all of the antigen sensitized groups. (b) Gene ontology (GO) biological processes significantly (p ≤ 0.05) enriched with the genes from BLG OVA and PNA sensitized groups. A total of 46 overlapping biological processes were commonly enriched among all 3 allergen sensitized groups.

Analysis of the differentially expressed genes was carried out on the MLN of BALB/c mice sensitized with common food antigens of varying allergenic potencies. These MLNs were collected 24 h after the second sensitization. Analysis of BLG sensitized mice revealed 1361 differentially expressed genes with 1030 up-regulated and 331 down-regulated (p < 0.05). A total of 533 genes were differentially expressed in the OVA sensitized group (p < 0.05); 256 genes were up-regulated and 277 genes were down-regulated. Finally, in PNA sensitized animals, 488 genes were differentially expressed (p < 0.05); 207 genes being up-regulated and 281 genes being down-regulated. The number of common and uniquely expressed genes for each antigen sensitization is indicated in Figure 1(a). A complete list of genes that were differentially expressed in response to BLG, OVA and PNA sensitization is provided in Additional file 1. A total of 150 genes were commonly expressed in all of the BLG, OVA, and PNA sensitized groups (Figure 1a; Additional file 2). Top 20 of these common differentially expressed genes are summarized in Table 1. As expected, sensitization with PBS did not have any significant effect on MLN gene expression.

**Table 1:** List of common top 10 up-regulated and top 10 down-regulated genes significantly (p < 0.05) differentially expressed in BALB/c mice in response to BLG, OVA and PNA sensitizations.

			BLG		OVA		PNA	
			
Unigene ID	Gene Symbol	Gene Name	Fold Change	P-Value	Fold Change	P-Value	Fold Change	P-Value
Mm.8245	Timp1	Tissue inhibitor of metalloproteinase 1	9.8	4E-03	18.4	2E-03	13.4	1E-02

Mm.259609	Prrg4	Proline rich Gla (G-carboxyglutamic acid) 4	3.5	3E-04	7.6	8E-03	4.7	1E-02

Mm.289645	Meg3	Maternally expressed 3	4.7	1E-03	4.2	1E-02	8.3	1E-02

Mm.646	Tpm2	Tropomyosin 2, beta	3.8	4E-05	2.6	4E-04	2.9	3E-04

Mm.295533	Flna	Filamin, alpha	3.2	3E-05	2.6	2E-04	2.4	8E-04

Mm.22701	Gas1	Growth arrest specific 1	3.2	2E-04	2.6	3E-04	2.1	7E-03

Mm.26700	Tmem16a	Transmembrane protein 16A	5.3	4E-03	2.5	3E-04	3.5	4E-03

Mm.12459	Ankrd10	Ankyrin repeat domain 10	3.5	4E-03	2.2	3E-03	2.6	7E-03

Mm.196110	Hba-a1	Hemoglobin alpha, adult chain 1	3.6	2E-03	2.2	5E-03	2.8	4E-03

Mm.20954	Rgs5	Regulator of G-protein signalling 5	2.8	9E-06	2.2	2E-04	2.5	2E-03

Mm.431947	Ovgp1	Oviductal glycoprotein 1	-4.4	4E-05	-4.6	1E-03	-3.0	2E-03

Mm.33401	Mgam	Maltase-glucoamylase	-5.5	6E-08	-4.6	2E-08	-9.2	6E-06

Mm.1641	Car4	Carbonic anhydrase 4	-3.6	1E-04	-4.7	6E-04	-8.0	7E-06

Mm.4533	Apoa4	Apolipoprotein A-IV	-11.2	5E-06	-5.3	2E-08	-19.1	8E-06

Mm.1186	Car2	Carbonic anhydrase 2	-1.5	1E-02	-6.0	2E-03	-5.2	7E-06

Mm.210336	Lgals4	Lectin, galactose binding, soluble 4	-11.2	8E-07	-7.2	2E-05	-5.0	3E-05

Mm.7244	Agr2	Anterior gradient 2	-5.5	3E-10	-9.7	9E-07	-13.9	7E-07

Mm.297976	Gpc1	Glypican 1	-4.9	7E-08	-10.7	3E-12	-7.3	1E-05

Mm.273195	Car1	Carbonic anhydrase 1	-5.4	8E-08	-12.6	2E-10	-8.2	9E-06

Mm.11869	AI427122	Expressed sequence AI427122	-8.3	4E-07	-13.2	2E-08	-8.5	2E-06

### Gene ontology (GO) analysis

**Figure 2:**
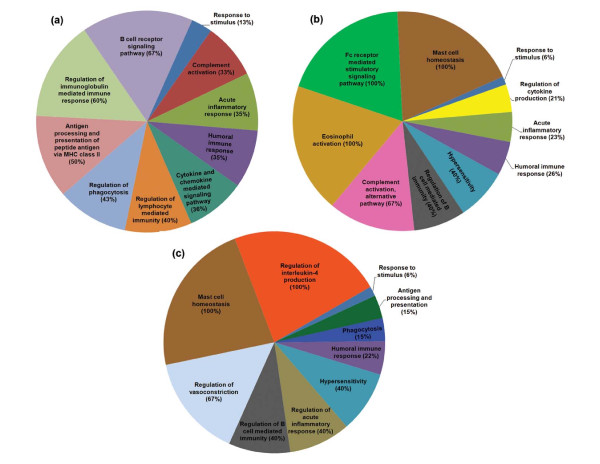
**Immune or hypersensitivity related GO biological processes significantly (p < 0.05) enriched with the genes from (a) BLG, (b) OVA and (c) PNA sensitized group with high percent (%) enrichment**. Numbers within the parenthesis indicate the percent enrichment of each GO biological process.

Genes that were significantly differentially expressed in response to antigen sensitization were further analyzed to understand their biological relevance and molecular functions. A total of 204 GO biological processes were identified as significantly enriched with the genes from BLG sensitized group (p < 0.05) (Figure 1b; Additional file 3). From these 204 biological processes, approximately 70 processes were directly related with immune or inflammatory response. Some of these immune response related biological processes with high percent (%) enrichment includes B cell receptor signaling pathway [GO:0050853], regulation of immunoglobulin mediated immune response [GO:0002889], antigen processing and presentation of peptide antigen via MHC class II [GO:0002495] and regulation of phagocytosis [GO:0050764] (Figure 2a). Genes differentially expressed in response to OVA sensitization enriched 327 GO biological processes (p < 0.05) (Figure 1b; Additional file 4), of which at least 71 processes were related with immune or hypersensitivity response. Some of these immune or hypersensitivity related processes high percent (%) enrichment includes mast cell homeostasis [GO:0033023], Fc receptor mediated stimulatory signaling pathway [GO:0002431], eosinophil activation [GO:0043307], complement activation, alternative pathway [GO:0006957], regulation of B cell mediated immunity [GO:0002712] and hypersensitivity [GO:0002524] (Figure 2b). Finally, GO analysis with the genes differentially expressed in response to PNA sensitization enriched 295 biological processes (p < 0.05) (Figure 1b; Additional file 5). Among these 295 biological processes, approximately 73 processes were immune or hypersensitivity related including regulation of interleukin-4 production [GO:0032673], mast cell homeostasis [GO:0033023], regulation of vasoconstriction [GO:0019229], regulation of B cell mediated immunity [GO:0002712], regulation of acute inflammatory response [GO:0002673] and hypersensitivity [GO:0002524] (Figure 2c). Comparison of the GO biological processes among allergen sensitized groups revealed a total of 46 overlapping biological processes commonly enriched (p < 0.05) in response to BLG, OVA and PNA sensitizations (Additional file 6). Further analysis revealed that almost 48% of these common processes (22 processes) were related to immune or hypersensitivity responses (Table 2). Two common differentially expressed genes, Fc receptor, IgE, high affinity I, gamma polypeptide (FcεR1γ) and Complement component 3 (C3) played a central role for the enrichment of the majority of these hypersensitivity or immune responsive biological processes (Table 3). Other genes that were involved in the enrichment of these common GO biological processes include Immunoglobulin joining chain (Igj), Keratin 8 (Krt8), Scinderin (Scin), Signal transducer and activator of transcription 3 (Stat3), Supervillin (Svil) and Villin 1 (Vil1) (Table 3).

**Table 2:** Gene ontology (GO) biological processes related with immune, inflammatory or hypersensitivity responses commonly enriched with the genes differentially expressed in BALB/c mice in response to BLG, OVA and PNA sensitizations.

		BLG Sensitized		OVA Sensitized		PNA Sensitized	
		
GO Biological Processes	GO ID	Genes	Fisher's Exact P-Value	Genes	Fisher's Exact P-Value	Genes	Fisher's Exact P-Value
Response to stimulus	50896	100	0.005	50	0.011	50	0.002

Actin filament-based process	30029	22	0.011	11	0.041	12	0.009

Cytokine and chemokine mediated signaling pathway	19221	10	0.000	4	0.038	5	0.006

Acute inflammatory response	2526	9	0.001	6	0.001	5	0.004

Humoral immune response	6959	8	0.001	6	0.001	5	0.002

Regulation of actin filament depolymerization	30834	6	0.011	4	0.012	5	0.001

Actin filament depolymerization	30042	6	0.011	4	0.012	5	0.001

Humoral immune response mediated by circulating immunoglobulin	2455	6	0.001	3	0.024	3	0.019

Negative regulation of actin filament depolymerization	30835	5	0.028	4	0.008	5	0.001

Phagocytosis	6909	5	0.043	4	0.012	3	0.049

Positive regulation of defense response	31349	4	0.044	4	0.003	3	0.019

Positive regulation of endocytosis	45807	4	0.025	3	0.016	3	0.012

Positive regulation of response to external stimulus	32103	4	0.034	3	0.020	3	0.015

Positive regulation of inflammatory response	50729	3	0.038	3	0.005	3	0.004

Myeloid leukocyte mediated immunity	2444	3	0.026	3	0.003	2	0.032

Positive regulation of phagocytosis	50766	3	0.026	2	0.038	2	0.032

Regulation of phagocytosis	50764	3	0.026	2	0.038	2	0.032

Positive regulation of immunoglobulin mediated immune response	2891	3	0.009	2	0.019	2	0.016

Regulation of immunoglobulin mediated immune response	2889	3	0.009	2	0.019	2	0.016

Positive regulation of inflammatory response to antigenic stimulus	2863	3	0.016	2	0.028	2	0.023

Positive regulation of B cell mediated immunity	2714	3	0.009	2	0.019	2	0.016

Regulation of B cell mediated immunity	2712	3	0.009	2	0.019	2	0.016

**Table 3:** Some significant genes involved in the enrichment of the common GO biological processes related to immune or hypersensitivity.

		Gene Ontology Biological Process		
		
UniGene ID	Gene Symbol	BLG Sensitized Group	OVA Sensitized Group	PNA Sensitized Group
Mm.19131	C3	Acute inflammatory response [GO:0002526]; Humoral immune response [GO:0006959]; Phagocytosis [GO:0006909]; Positive regulation of response to external stimulus [GO:0032103]; Regulation of B cell mediated immunity [GO:0002712]; Positive regulation of defense response [GO:0031349]	Acute inflammatory response [GO:0002526]; Humoral immune response [GO:0006959]; Phagocytosis [GO:0006909]; Positive regulation of response to external stimulus [GO:0032103]; Regulation of B cell mediated immunity [GO:0002712]; Positive regulation of defense response [GO:0031349]	Acute inflammatory response [GO:0002526]; Humoral immune response [GO:0006959]; Phagocytosis [GO:0006909]; Positive regulation of response to external stimulus [GO:0032103]; Regulation of B cell mediated immunity [GO:0002712]; Positive regulation of defense response [GO:0031349]

Mm.22673	Fcer1g	Acute inflammatory response [GO:0002526]; Phagocytosis [GO:0006909]; Positive regulation of response to external stimulus [GO:0032103]; Regulation of B cell mediated immunity [GO:0002712]; Positive regulation of defense response [GO:0031349]	Acute inflammatory response [GO:0002526]; Phagocytosis [GO:0006909]; Positive regulation of response to external stimulus [GO:0032103]; Regulation of B cell mediated immunity [GO:0002712]; Positive regulation of defense response [GO:0031349]	Acute inflammatory response [GO:0002526]; Phagocytosis [GO:0006909]; Positive regulation of response to external stimulus [GO:0032103]; Regulation of B cell mediated immunity [GO:0002712]; Positive regulation of defense response [GO:0031349]

Mm.342177	Igh-6	Humoral immune response [GO:0006959]	Humoral immune response [GO:0006959]	Humoral immune response [GO:0006959]

Mm.1192	Igj	Humoral immune response [GO:0006959]	Humoral immune response [GO:0006959]	Humoral immune response [GO:0006959]

Mm.358618	Krt8	Cytokine and chemokine mediated signaling pathway [GO:0019221]	Cytokine and chemokine mediated signaling pathway [GO:0019221]	Cytokine and chemokine mediated signaling pathway [GO:0019221]

Mm.2416	Scin	Regulation of actin filament depolymerization [GO:0030834]	Regulation of actin filament depolymerization [GO:0030834]	Regulation of actin filament depolymerization [GO:0030834]

Mm.249934	Stat3	Cytokine and chemokine mediated signaling pathway [GO:0019221]; Acute inflammatory response [GO:0002526]	Acute inflammatory response [GO:0002526]; Cytokine and chemokine mediated signaling pathway [GO:0019221]	Cytokine and chemokine mediated signaling pathway [GO:0019221]; Acute inflammatory response [GO:0002526]

Mm.136791	Svil	Regulation of actin filament depolymerization [GO:0030834]	Regulation of actin filament depolymerization [GO:0030834]	Regulation of actin filament depolymerization [GO:0030834]

Mm.471601	Vil1	Regulation of actin filament depolymerization [GO:0030834]	Regulation of actin filament depolymerization [GO:0030834]	Regulation of actin filament depolymerization [GO:0030834]

GO analysis also revealed the molecular functions of the differentially expressed genes. A total of seven GO molecular functions were commonly enriched with the genes from BLG, OVA and PNA sensitized groups (Table 4). Among these molecular functions calcium ion binding [GO:0005509] enriched with the highest number of genes from either BLG, OVA or PNA sensitized groups. Actin binding [GO:0003779] is another significant commonly enriched molecular function; similar actin filament related processes were also observed to be enriched in GO biological process category (Table 2).

**Table 4:** Common GO molecular functions enriched with the genes differentially expressed in BALB/c mice in response to BLG, OVA and PNA sensitizations.

		BLG Sensitized		OVA Sensitized		PNA Sensitized	
		
GO Molecular Functions	GO ID	Genes	Fisher's Exact P-Value	Genes	Fisher's Exact P-Value	Genes	Fisher's Exact P-Value
Calcium ion binding	5509	58	0.0003	34	0.0001	36	<0.0001

Cytoskeletal protein binding	8092	39	0.002	22	0.0019	23	0.0002

Actin binding	3779	30	0.0024	18	0.0012	20	<0.0001

Oxidoreductase activity, acting on CH-OH group of donors	16614	14	0.0074	17	<0.0001	9	0.0022

Structural constituent of cytoskeleton	5200	11	0.0005	6	0.0055	9	<0.0001

Antigen binding	3823	3	0.0085	3	0.0009	3	0.0007

Structural constituent of muscle	8307	3	0.0037	2	0.0119	2	0.0099

### Validation of the differentially expressed genes by real-time RT-PCR

**Figure 3:**
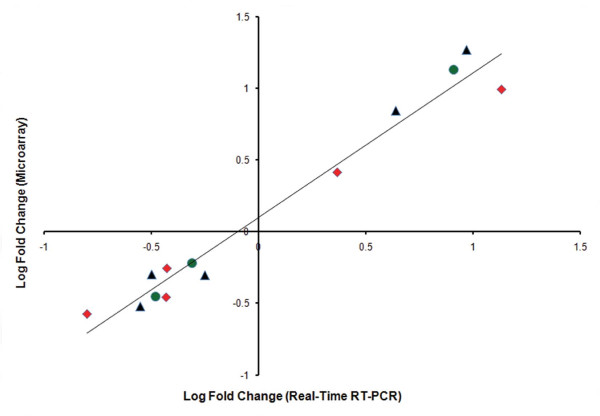
**Correlation of the corresponding fold changes of the validated genes from microarray and real-time RT-PCR experiments**. Genes validated from BLG sensitized group are Stard4, Igj, Timp1, Cd79a and Itgb1, and indicated with a "red diamond". Genes validated from OVA sensitized group are Stard4, Igj, Timp1, Pex13 and Syt4 and indicated with a "black triangle". Genes validated from PNA sensitized group are Stard4, Igj and Timp1, and indicated with a "green circle". Genes Stard4, Igj and Timp1 were commonly expressed in response to all three antigen sensitizations. Genes Cd79a and Itgb1 were only expressed in BLG sensitized mice, and genes Pex13 and Syt4 were expressed only in OVA sensitized mice.

Differential expression of 7 representative genes from the microarray experiment was validated by real-time RT-PCR. These seven genes were selected based on their previous known relation with allergy, fold change, and p-values. These genes included Stard4 (StAR-related lipid transfer domain containing 4), Igj (Immunoglobulin joining chain), Timp1 (Tissue inhibitor of metalloproteinase 1), Cd79a (CD79A antigen), Itgb1 (Integrin beta 1 or fibronectin receptor beta), Pex13 (Peroxisomal biogenesis factor 13) and Syt4 (Synaptotagmin IV) (Table 5). The corresponding real-time RT-PCR and microarray fold changes in gene expression were highly correlated for all of the seven validated genes (Figure 3), as indicated by Spearman's rank correlation rho value of 0.923 (p < 0.001).

**Table 5:** Representative genes from microarray experiment validated by real-time RT-PCR with their corresponding fold changes and p-values.

	Microarray Experiment						Real Time RT-PCR Experiment					
	
Gene Symbol	BLG Sensitization		OVA Sensitization		PNA Sensitization		BLG Sensitization		OVA Sensitization		PNA Sensitization	
	
	Fold Change	P-Value	Fold Change	P-Value	Fold Change	P-Value	Fold Change	P-Value	Fold Change	P-Value	Fold Change	P-Value
**Stard4**	-2.9	6E-04	-3.3	2E-03	-2.8	2E-04	-2.7	1E-03	-3.6	1E-03	-3.0	1E-03

**Igj**	-1.8	8E-05	-2.0	1E-06	-1.7	7E-05	-2.7	2E-02	-3.1	2E-02	-2.0	4E-02

**Timp1**	9.8	4E-03	18.4	2E-03	13.4	1E-02	13.6	1E-02	9.4	3E-03	8.1	3E-02

**Cd79a**	-3.7	5E-03	-	-	-	-	-6.3	4E-04	-	-	-	-

**Itgb1**	2.6	7E-03	-	-	-	-	2.3	4E-02	-	-	-	-

**Pex13**	-	-	-2.0	5E-03	-	-	-	-	-1.8	3E-02	-	-

**Syt4**	-	-	7.0	8E-03	-	-	-	-	4.4	3E-02	-	-

### Topical ear challenges

**Figure 4:**
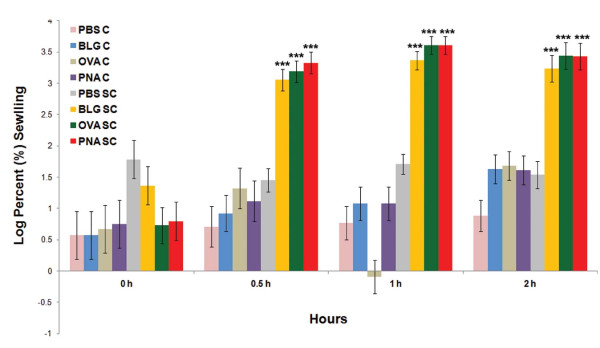
**Ear swelling response in BALB/c mice**. Percent (%) ear swelling was measured over time in BALB/c mice in response to BLG (n = 8), OVA (n = 8) or PNA (n = 8) sensitization and challenge (SC). Mice in the BLG, OVA and PNA SC groups indicated a significant ear swelling response when compared to that in PBS SC mice (n = 8) or mice in the BLG (n = 4), OVA (n = 4), PNA (n = 4) and PBS (n = 4) challenge (C) only groups. Results expressed as LSM ± standard error of the mean (SEM). *** represents p < 0.001.

The ear swelling response was measured in BALB/c mice to assess the type I hypersensitivity response induced by the food allergens. All mice that were sensitized and challenged with 2% BLG, 2% OVA or 0.2% PNA responded significantly with an ear swelling response when compared to mice that received only PBS, or were challenged only with antigen (p < 0.001). Linear and quadratic contrasts revealed that the ear swelling among mice that were sensitized and challenged with BLG, OVA and PNA antigens increased significantly at all time points (p < 0.001). The peak ear swelling was recorded at 1 h post challenge and the response was gradually deceased over time (Figure 4). Figure 4 indicates ear swelling response only at 0.5, 1 and 2 h post-challenge. The ear swelling responses were not significantly different among antigen treatments (Additional file 7).

### Total serum IgG1 and IgE response to sensitization and topical challenge

**Figure 5:**
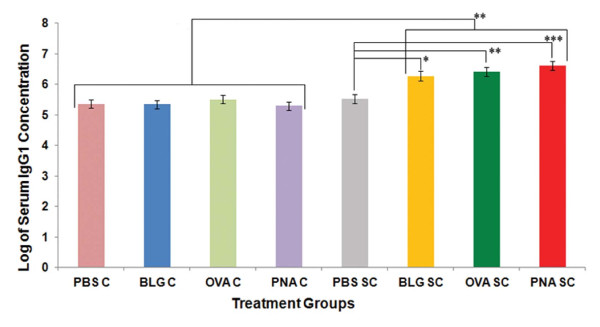
**Serum total IgG1 concentrations in BALB/c mice 24 h post challenge**. Results are expressed as LSM ± SEM. Multiple comparisons among the treatments within groups were performed using Bonferroni's correction to adjust the p-values for the type-1 error rate. * p < 0.02; ** p < 0.01; *** p < 0.002

**Figure 6:**
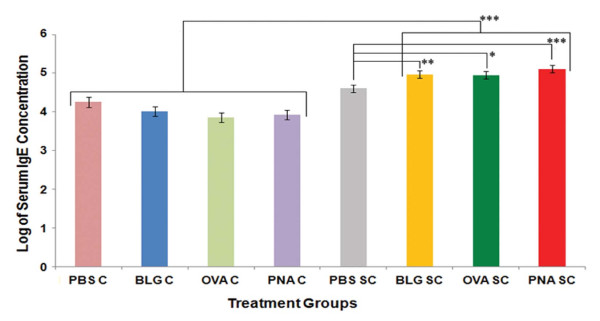
**Serum total IgE concentrations in BALB/c mice 24 h post challenge**. Results are expressed as LSM ± SEM. Multiple comparisons among the treatments within groups were performed using Bonferroni's correction to adjust the p-values for the type-1 error rate. * p < 0.06; ** p < 0.05; *** p < 0.01

Immunogenicity and allergenicity of each antigen was evaluated by measuring changes in total serum IgG1 and IgE concentrations respectively 24 h post challenge. Total IgG1 concentrations in mice that were sensitized and challenged with BLG, OVA or PNA increased when compared to mice that received PBS alone, or were challenged only with antigens (p < 0.02; Figure 5). Total IgE concentrations in the serum were also significantly increased in animals that had been sensitized and challenged with BLG and PNA when compared to mice that received only PBS or were challenged with antigen, alone (p < 0.05; Figure 6). Concentrations of IgE in the OVA treated mice were also increased, but statistical significance was not achieved after Bonferroni's correction (p = 0.06). IgG1 and IgE concentrations were not significantly different among antigen treatments.

### Plasma histamine concentrations in antigen sensitized and i.p. challenged mice

Plasma histamine concentrations were measured as an indicator of mast cell degranulation in BALB/c mice that had been sensitized and challenged with BLG, OVA or PNA. Elevated plasma histamine concentrations were observed in OVA and PNA sensitized and challenged mice, when compared to mice that received only PBS (p < 0.01; Table 6). Histamine concentrations were also increased in mice from the BLG treatment, however, this did not reach statistical significance (p = 0.06). Histamine concentrations among antigen treatment groups were significantly different (p < 0.01).

**Table 6:** Plasma histamine concentrations in BALB/c mice after allergen treatment. BALB/c mice i.p. sensitized and challenged with BLG, OVA and PNA had a rapid histamine induction 20 min after the challenge. * p = 0.06; ** p < 0.01

Treatment Group	Treatment Type	Plasma Histamine Concentration (ng/ml)	Plasma Histamine Level (LSM ± SEM)
PBS Sensitized and Challenged	Control	24.12	2.96 ± 0.27

BLG Sensitized and Challenged	Allergy	67.64	4.06 ± 0.27*

OVA Sensitized and Challenged	Allergy	567.48	6.31 ± 0.27**

PNA Sensitized and Challenged	Allergy	334.15	5.76 ± 0.27**

### Passive cutaneous anaphylaxis (PCA) reaction

A PCA assay was carried out to evaluate the antigen-specific IgE response in BALB/c mice that had been sensitized and challenged with BLG, OVA or PNA. Results from PCA assay indicated that antigen-specific serum from previously exposed mice was sufficient to elicit a type I hypersensitivity response in naïve mice (p < 0.05). Seventy five percent of mice that received BLG or OVA-specific anti-serum had a positive PCA response, whereas 100% mice that received PNA specific anti-serum had a positive PCA response (Table 7). As expected, none of the mice in any of the control groups had any positive PCA reaction.

**Table 7:** Passive Cutaneous Anaphylaxis (PCA) assay in BALB/c mice. A blue wheal of a diameter of 3 mm or more, 30 min following challenge was considered as positive response and indicative of the presence of allergen-specific IgE. * p < 0.05

	Treatment		Diameter (mm)	Positive Reaction
	
Groups	Sensitization	Allergen Challenge	Mean ± SEM	(n) Responder/Total
	Naïve Serum	PNA	0.9 ± 0.07	0/3
	
**Naïve Control**	Naïve Serum	OVA	0.8 ± 0.15	0/3
	
	Naïve Serum	BLG	1.0 ± 0.03	0/3

	PBS SC Serum	PNA	1.1 ± 0.13	0/3
	
**PBS Control**	PBS SC Serum	OVA	1.1 ± 0.21	0/3
	
	PBS SC Serum	BLG	1.4 ± 0.26	0/3

**Experimental Group 1**	PNA SC Serum	PNA	5.1 ± 0.43*	4/4

**Experimental Group 2**	OVA SC Serum	OVA	4.5 ± 0.74*	3/4

**Experimental Group 3**	BLG SC Serum	BLG	4.1 ± 0.72*	3/4

## Discussion

In this study, BALB/c mice were used as a model of food allergy in order to analyze the MLN transcriptome profiles to explore the molecular mechanism(s) associated with sensitization to three of the most common food allergens; BLG, OVA and PNA. Analysis of ear swelling, plasma histamine, total IgG1 and IgE concentrations, and antigen-specific IgE level (PCA) indicated that BALB/c mice developed a type I allergic response to these food allergens. These immunological profiles also indicate that the initial exposure doses without any adjuvants were sufficient to induce allergen sensitization in BALB/c mice.

Although the ear swelling response is typically used to assess contact allergy (type IV hypersensitivity) [31], tape-stripping prior to topical antigenic challenge enabled us to use this endpoint to measure a type I hypersensitivity response. Massive ear swelling was observed as early as 30 min post challenge and peaked 1 h post challenge. Such rapid swelling is characteristic of IgE-mediated mast cell degranulation, a hallmark of a type I allergic response [32]. The ear swelling test also proved to be sensitive and was able to differentiate the potencies of the antigens that used in this study. For example, the ear swelling data demonstrated that PNA was more potent than BLG and OVA for eliciting a type I allergic response. The concentration of PNA solution (0.2%) that used in this study was 10 times less than the concentrations used for BLG and OVA solution (2%), yet the magnitude of ear swelling observed in PNA sensitized and challenged mice was not significantly different to that observed in BLG or OVA sensitized and challenged mice (Additional file 7). These results demonstrate that this method is capable of differentiating the antigen potency and also able to generate reliable quantitative data for assessing the magnitude a type I allergic response.

IgE antibodies are known to play a central role in mediating type I hypersensitivity reactions. In our study, increased production of IgE in BALB/c mice sensitized and challenged with BLG, OVA or PNA illustrates that these mice experienced a IgE mediated type I allergic reaction. In order to confirm that BALB/c mice used in this study had an IgE mediated type I allergic response and anaphylaxis, plasma histamine concentrations were measured. In this study, rapid release of histamine within 20 min of the i.p. challenge confirmed the induction of type I allergic response and subsequent degranulation of mast cells. Traditionally, it was believed that food allergy-related systemic anaphylaxis was only due to the interaction between IgE and FcεRI on the mast cell surface, and this lead to mast cell degranulation and the subsequent release of histamines and other enzymes [33]. Reports, however, suggest that an alternate pathway exists that is dependent on the interaction of IgG1 and FcγRIII [34]. Mouse FcγRIII is a low affinity receptor, present on the surface of the macrophages, neutrophils and natural killer cells that binds to IgG1. Binding of soluble IgG1 with blood-borne antigens must occur before they bind to macrophage FcγRIII. In contrast to the IgE-mediated pathway, this pathway takes relatively longer to induce anaphylaxis, requires high concentrations of IgG1 for elicitation, and involves platelet activating factors (PAF), rather than histamine, as the primary mediator in this type of anaphylactic shock [34,35]. In our study, the release of histamine within 20 min of i.p. challenge of BALB/c mice strongly suggests that allergic and anaphylactic responses related to BLG, OVA or PNA sensitization and challenge were IgE mediated.

Finally, the PCA data in this study demonstrated that BALB/c mice responded positively and developed BLG-, OVA- or PNA-specific immunoglobulin IgE. This positive PCA response was exclusively due to the binding of antigen-specific IgEs with FcεRIs on mast cell rather than the binding of IgG1 with FcγRIII, since mice were sensitized with antigen specific anti-sera and challenged with the respective antigens and Evans blue dye after 48 h of anti-sera sensitization; only IgE is capable of remain bound to high affinity FcεRI for this period of time [22].

Analysis of MLN transcriptome profile of BALB/c mice sensitized with BLG, OVA or PNA revealed the differential expression of numerous genes that are associated with activation of the Th2 response. This method of transcriptome profiling appeared to be very sensitive that identified differential expression of hundreds of genes in BALB/c mice within 24 h of the second sensitization with the given food allergens. Comparison of the differentially expressed genes in BLG, OVA and PNA sensitized groups implicates that a common set of genes involved in the sensitization phase of the immune response to these three food allergens in BALB/c mice. A total of 150 overlapping genes being differentially expressed in the same direction among the three food allergen sensitized groups. Among these genes, the expression of Timp1 was up-regulated, whereas the expression of Stard4 and Igj were down-regulated in all three antigen sensitized groups. The involvement of Timp1 with the allergic response is very well documented; being an inhibitor of matrix metalloproteinases, it plays a central role in degradation of extracellular matrix and remodeling of tissue during inflammatory process [36], and appears to be involved in asthma [37,38]. Induction of Timp1 has also been implicated in the pathogenesis of other allergic conditions including atopic dermatitis, allergic contact dermatitis and chronic obstructive pulmonary disease (COPD) [36,39,40].

Stard4 is known to be involved in the binding and directional transport of the cholesterol into the liver for the synthesis of bile acid [41], and its expression is reported to be down-regulated in the presence of cholesterol [42]. It is not clear from available reports whether Stard4 plays any direct role in the allergic response, however, the microarray and real-time RT-PCR data from all antigen sensitized groups in our study demonstrates its involvement in the sensitization of food allergens.

Igj gene products are produced by antibody secreting plasma cells after antigen or cytokine stimulation. They are incorporated into pentameric IgM and dimeric IgA immunoglobulins and play a crucial role in the cellular and mucosal secretion of these molecules [43-45]. Plager *et al. *[43] reported that the expression of Igj is down-regulated in patients with atopic dermatitis and this may be due to isotype switching of plasma cells from IgM and IgA production to IgG1 and IgE production. Down-regulation of Igj in both microarray and real-time RT-PCR experiments in our study among all antigen sensitized treatments indicates a possible induction of type I allergic reaction, and this was supported by the endpoints that we measured during the challenge phase.

Several uniquely expressed genes were also activated in response to BLG, OVA or PNA sensitization. For example, Itgb1 was up-regulated by BLG sensitization; this plasma membrane receptor is expressed on lymphocytes, monocytes and eosinophils, and may be involved in inducing allergic inflammatory response [46,47]. Available reports also suggest that Itgb1 may be involved in the airway smooth muscle responsiveness through the association with fibronectin and type I collagen during asthma or allergic inflammation [48]. The observed up-regulation of Itgb1, as indicated by both microarray analysis and real-time RT-PCR, is in agreement with these available reports, and demonstrates its possible role in food allergic process. In contrast, CD79a was down-regulated in BLG sensitized group, and genes Syt4 and Pex13 were up- and down-regulated, respectively in OVA sensitized group. It is not known whether the genes CD79a, Syt4 and Pex13 play any direct role in any allergic conditions. However, microarray analysis and real-time RT-PCR results from this study implicates their involvement in BLG or OVA sensitization.

Gene ontology analysis was also used to reveal the biological roles and molecular functions of the differentially expressed genes that were commonly and uniquely expressed among the allergen sensitized mice. These enriched GO data further validated the involvement of the differentially expressed genes in allergen sensitizations in BALB/c mice. A total of 46 GO biological processes were commonly enriched among all three antigen sensitized groups (p ≤ 0.05) and at least 22 of these processes were related to immune or hypersensitivity related processes (Table 2). The most significant overlapping enriched GO biological process was the response to stimulus [GO:0050896]; this process was enriched with 50 genes from the OVA and PNA sensitized groups, and with 100 genes from BLG sensitized group (Table 2). Within the response to stimulus biological process, was a subcategory biological process referred to as immune response [GO:0006955]. A total of 30 genes from BLG, 12 genes from OVA and 13 genes from PNA sensitized groups enriched this biological process. In addition to the immune response biological process other biological processes that were subcategories within the response to stimulus biological process were enriched with genes from BLG, OVA and PNA sensitized groups. These subcategories included the acute inflammatory response [GO:0002526], humoral immune response [GO:0006959], regulation of immunoglobulin mediated immune response [GO:0002889] and regulation of B cell-mediated immunity [GO:0002712]. Enrichment of these biological processes indicates that the genes differentially expressed in response to BLG, OVA or PNA sensitization are involved in the process of immune regulation or hypersensitivity responses.

Two common differentially expressed genes that were involved in the enrichment of several of these immune or hypersensitivity related biological processes were Fcer1γ and C3 (Table 3), whose expression was up-regulated in all of the allergen treated groups of BALB/c mice. Among other genes, Immunoglobulin heavy chain complex (Igh-6), and Signal transducer and activator of transcription 3 (Stat3), and Immunoglobulin joining chain (Igj) also played important role in the enrichment of other immune or hypersensitivity related biological processes (Table 3). The involvement of FcεR1γ, Stat3 and C3 with the allergic responses are very well documented. FcεR1γ for example, is expressed on the surface of mast cell and cross-linking of these IgE bound receptors leads to mast cell degranulation, cytokine production, prostaglandin synthesis, survival and passive systemic anaphylaxis [49]. It has also been reported that elevated level of transcription factor Stat3 is associated with the induction of house dust mite-mediated allergic inflammation and airway hyper-responsiveness in mice [50]. Likewise, Stat3 is required for the active production of IL-21-mediated IgE production by human B cells [51]. Complement C3 plays a central role as a mediator of airway hyper-responsiveness and asthma, and induces the expression of the Th2 phenotype [52]. Anaphylatoxin C3a, an enzymatic derivative of C3 also acts as a chemotactic factor that triggers the release of histamines and cationic proteins from mast cells and eosinophils respectively [53]. C3a also plays a role in smooth muscle contraction that is mediated by leukotrienes, prostanoids, and platelet-activating factor released from mast cells and eosinophils [53]. Other commonly enriched GO biological processes include actin filament-based process [GO:0030029] and muscle development process [GO:0007517]. Enrichment of these two biological processes suggests possible involvement of some of the differentially expressed genes with smooth muscle contraction, bronchoconstriction and vasodilation that are the common phenomena associated with type I allergic responses or anaphylaxis. Some of the differentially expressed genes enriched these processes include Scinderin (Scin), Supervillin (Svil) and Villin 1 (Vil1). Scin is an actin-filament severing and capping protein activated by calcium is known to be differentially expressed in BALB/c mice exposed to OVA, and suggested to be a potential biomarker of asthma, a type I allergy [54]. Similarly, Vil1 is another actin-binding protein that is known for IgE-binding and IgE-cross-reactivity with other plant proteins of the same family [55]. Therefore, differential expression of these genes and enrichment of these GO biological processes illustrate the possible involvement of these genes in food allergic sensitizations.

GO analysis further revealed the molecular functions of some of the differentially expressed genes (Table 4). One significant overlapping enriched GO molecular function was the calcium ion binding [GO: 0005509]. Enrichment of this molecular function is well correlated with other enriched biological processes and emphasized its involvement in the food allergy response. A recent report suggests that the binding of the antigens with the receptors on the T, B or mast cells induces a series of biochemical reactions leading to a transient increase in cytosolic free calcium concentration that leads to other downstream reactions in the process of allergic response [56]. Another report also suggests that antigen-induced FcεRI-mediated cell migration/chemotaxis is dependent on cytosolic free calcium concentration [57]. Therefore, the enrichment of these GO biological processes and molecular functions support the involvement of these differentially expressed genes in the food allergic responses during early sensitization phase. Thus, these gene expression data enabled us to monitor the allergenicity of some of the common food allergens and proved to be sensitive enough to identify the allergic/immune responses within 24 h of the sensitizations in the used animal model.

The experimental mice used in this study were systemically exposed to the food antigens by i.p. injection without any adjuvants, and they responded positively to all of the antigens, as indicated by the increased ear swelling response, elevated serum IgG1, IgE, plasma histamine and a positive PCA test. Oral sensitization was specifically avoided in this study because there is a potential risk of developing oral tolerance when antigens are administered via this route [25,29]. In order to avoid the development of oral tolerance, adjuvants, such as cholera toxin is being used during oral sensitization processes. However, immune/allergic response developed such a way does not mimic the actual immune/allergic response in humans and this may lead to misinterpretation of the data due to hyperstimulation of the immune system [26,27]. In our study, we sensitized the BALB/c mice via the i.p. route with only two consecutive injections of allergens on day 0 and 1 without any adjuvants. Use of this route for sensitization not only allowed us to avoid the use of any kind of adjuvants but also allowed us to observe the true effects of the allergens used without having any unwanted external stimulations or influences. Thus, the gene expression data presented here in our study corresponds only to the effects of the food allergens used for sensitizations that may mimic the actual allergic responses in humans. In this study, the PCA data indicated that one mouse in BLG and another mouse in the OVA treated group did not appear to be sensitized. Although they were a different group of animals than those used for the gene expression study, there is the possibility that non-responders may contribute to the variation in gene expression during the sensitization phase.

## Conclusion

Taken together, microarray data from this study implicate the involvement of many previously known genes as well as numerous novel genes in the pathogenesis of food allergy. This method of gene expression analysis proved to be a sensitive method in identifying the allergenicity of known food proteins. Results from this study will help to better understand the molecular mechanism(s) involved in the sensitization to food allergens and may help in developing new drug target for the therapy of food allergy in future. The results presented here in this study are only the first step and further studies are needed to establish this method for using as a screening tool to assess the allergenicity of other food proteins. With further studies in future, the genes differentially expressed here in this study may establish a pool of biomarker genes that can be used to determine the potential allergenicity of other food proteins of novel origin. Future studies may want to consider characterizing the effects of time and dose-response relationships among these allergens and their influence on gene expression, and investigate if this model is effective for identifying allergens with low potency. Further studies are also needed to observe the effects of a non-allergenic protein on the gene expression profiling of this animal model.

## Methods

### Animals

Six-to-eight week old female BALB/c mice were purchased from Charles River (Saint-Constant, QC, Canada). All mice were maintained on a peanut, egg and milk free diet at the Charles River and University of Guelph Central Animal facilities. Animals were maintained at 22-24°C at a relative humidity of 40-50%. The light-to-dark cycle was maintained at 12 h from 7 AM to 7 PM. All of the animal experiments were approved by the University of Guelph Animal Care Committee according the Canadian Council on Animal Care (CCAC) guidelines.

### Antigens (allergens) and reagents

Peanut agglutinin (PNA), ovalbumin (OVA) from hen's egg white, and β-lactoglobulin (BLG) from cow's milk were purchased from Sigma-Aldrich Canada (Oakville, ON). The antigen concentrations used in this study were primarily based on the previously published reports [20,29,30] and our own pilot study (data not reported).

### Sensitization of mice and harvesting of MLN

A group of female BALB/c mice (n = 24) were randomly divided into four groups and each mouse received BLG, OVA, PNA or PBS during sensitization. These mice were sensitized over two consecutive days by a daily intraperitoneal (i.p.) injection of 250 μl of filter sterilized antigen solution (2% BLG, 2% OVA or 0.2% PNA) in PBS or PBS. Mice were euthanized 24 h after the second i.p. injection using CO_2 _and MLNs were collected in cryogenic vials then snap frozen immediately in liquid nitrogen. Samples were stored at -70°C until total RNA was extracted.

### Preparation of reference cDNA for microarray analysis

Naïve female BALB/c mice (n = 10) were considered a common reference group for this study. Total RNA was extracted from the MLNs of these naïve mice, pooled, quantified and converted into cDNA as described below for use as common reference cDNA for microarray hybridization.

### Total RNA extraction

Total RNA was extracted from the MLN of individual mice using TRI Reagent (Ambion Inc., Austin, TX) according to the manufacturer's instruction. The extracted total RNA was further purified using the QIAGEN RNeasy Mini Kit (QIAGEN Canada Inc., Mississauga, ON) according to manufacturer's instruction. The quality and the concentration of the RNA samples were determined with an Agilent 2100 Bioanalyzer (Agilent Technologies, CA, USA) and a NanoDrop ND-1000 spectrophotometer (Thermo Fisher Scientific Inc., Wilmington, DE, USA).

### Preparation, purification and labeling of cDNA

First strand cDNA was prepared, purified and labeled using the SuperScript™ indirect cDNA labeling system kit (Invitrogen, Canada) according to the manufacturer's instruction. Concentrations of the purified pre- and post-labeled cDNA were determined with a NanoDrop^® ^ND-1000 spectrophotometer (Thermo Fisher Scientific Inc., Wilmington, DE, USA). For each microarray hybridization, equal amounts (~1 μg) of labeled common reference cDNA and labeled sample cDNA from individual mice sensitized with antigens (BLG, OVA or PNA) or PBS were thoroughly mixed by pipetting and concentrated to a volume of 12 μl using a speedvac at a high drying rate for 35-40 min. The final volume of the combined cDNA sample was brought to 120 μl with SlideHyb Hybridization Buffer # 1 (Ambion, TX, USA) and mixed thoroughly by pipetting before hybridization.

### Hybridization and scanning of the cDNA microarrays

Combined cDNA samples were hybridized to mouse 22.4 K cDNA arrays (University Health Network, Toronto, ON, Canada) that contained 22.4 K clone sets of mouse ESTs from the National Institute of Aging (NIA). For each of the antigen (BLG, OVA or PNA) or PBS sensitized groups, dye swapping was performed to minimize dye bias. Hybridization and washing was conducted using GeneTac HybStation (Genomic Solutions, MI, USA). Hybridization was continued for 18 h at 42°C with mild agitation. The arrays were washed with 3 different wash solutions immediately after hybridization. Wash 1 was carried out at 50°C with 5 cycles at a flowing time of 20 s and a holding time of 40 s using wash solution 1 (1 × SSC, 0.1% SDS). Washes 2 and 3 were carried out with wash solution 2 (0.1 × SSC, 0.1% SDS) and wash solution 3 (0.1 × SSC), respectively, at 40°C. The slides were immediately removed from the HybStation after washing and dried in a plastic tube by centrifugation at 400 × g for 2 min. Microarray slides were scanned using Axon 4200A scanner (Molecular Devices, CA, USA) and the images were recorded using GenePix Pro 6 software (Molecular Devices, PA, USA). Microarray data files from this study are available at EMBL-EBI ArrayExpress database (http://www.ebi.ac.uk/microarray-as/ae/). ArrayExpress accession number for these files is E-MEXP-2676.

### Analysis of microarray data

Microarray data was analyzed using GeneSpring GX 7.2 software (Agilent Technologies, CA, USA). Data were normalized by per spot, per chip: intensity dependent LoWeSS (locally weighted scatterplot smoothing) method [58]. A gene was considered to be expressed, if it had an intensity of >100. A gene was considered to be differentially expressed and statistically significant, when it had a fold change (expression ratio) of at least ± 1.5 with a p-value ≤ 0.05. Benjamini and Hochberg multiple testing correction was applied to minimize any false positive results. Gene Ontology (GO) analysis was carried out using GoMiner [59]. A GO biological process was considered to be enriched when the Fisher's exact p-value was ≤ 0.05.

### Real-time RT-PCR

Total RNA was reverse-transcribed using SuperScript™ III Reverse Trancriptase kit (Invitrogen Canada) according to manufacturer's instruction. Concentrations of cDNA were determined using Quant-iT™ PicoGreen^® ^dsDNA Kit (Molecular Probe, Invitrogen) according to manufacturer's instruction using a Victor 1420 Multilabel Counter machine (Perkin Elmer, MA, USA). Primers (Table 8) for the genes for the real-time RT-PCR experiment were generated using Primer Express software (Applied Biosystems Inc., CA, USA). Before conducting the real-time RT-PCR, regular PCR was performed on each of the selected gene using Taq DNA Polymerase (Invitrogen, Canada) on a GeneAmp PCR System 9700 machine (Applied Biosystems, CA, USA) to check the primer efficacy and to optimize the amount of template and primers to be used for real-time RT-PCR. Real-time RT-PCR was conducted using the Platinum^® ^SYBR^® ^Green qPCR SuperMix-UDG kit (Invitrogen Canada) according to manufacturer's instructions in an ABI Prism 7000 Sequence Detection System machine (Applied Biosystems Inc., CA, USA). The cDNA generated from common reference RNA samples from naïve mice (same RNA source used for microarray experiment) were used as negative control and RNA from the PBS treated group of mice were used as positive control in the real-time RT-PCR experiment. Standard curves were prepared from serially diluted cDNAs prepared by pooling cDNAs from all of the control and experimental (BLG, OVA or PNA sensitized) cDNA samples. Data generated from real-time RT-PCR were analyzed by standard curve method for relative quantification of gene expression [60]. All real-time RT-PCR data were normalized relative to an internal control/housekeeping gene (Ribosomal protein L13a; Rpl13a). A Student's t-test was performed to determine the statistical significance of the analyzed genes and p-values of ≤ 0.05 were considered to be statistically significant. A correlation analysis between the real-time RT-PCR and microarray fold changes of the validated genes was performed using the Spearman's rank correlation analysis. A Shapiro-Wilk normality test for the fold change dataset was performed prior to the correlation analysis.

**Table 8:** Primers for the real-time RT-PCR experiment. Sequences of the forward and reverse primers of the differentially expressed and housekeeping genes for the real-time RT-PCR experiment.

Accession ID	Gene Symbol	Direction	Primer Sequence
**House Keeping Gene**			

Mm.180458	Rpl13a	Forward	5' GAGAAACGGAAGGAAAAGGCC

Mm.180458	Rpl13a	Reverse	5' GCAGGCATGAGGCAAACAGT

**Differentially Expressed Genes**			

Mm.127058	Stard4	Forward	5' GAGTGGCGAGTTGCCAAAAA

Mm.127058	Stard4	Reverse	5' CCACGTCATCCATAACTCCTTGA

Mm.1192	Igj	Forward	5' TCATCCCTTCCACCGAGGA

Mm.1192	Igj	Reverse	5' CCAGCTCCACTTCCACAGGA

Mm.8245	Timp1	Forward	5' AAATGCCGCAGATATCCGGT

Mm.8245	Timp1	Reverse	5' CTGATGTGCAAATTTCCGTTCC

Mm.1355	Cd79a	Forward	5' ACAGGCCAGCTGTTCTTCCC

Mm.1355	Cd79a	Reverse	5' GCTGTGATGATGCGGTTCTTG

Mm.263396	Itgb1	Forward	5' CCAGTCCCAAGTGCCATGAG

Mm.263396	Itgb1	Reverse	5' GCAGTAAGCGTCCATGTCTTCAC

Mm.286622	Pex13	Forward	5' GTGGCCTGCCTTAGTGCTGA

Mm.286622	Pex13	Reverse	5' CGTGGTCATCCTCACCACTTG

Mm.233846	Syt4	Forward	5' CTTATCCCCACATCCAAGAGCTC

Mm.233846	Syt4	Reverse	5' AGACCAGAAGTTCACCCCGG

### Antigen sensitization and topical ear challenge

Another group of BALB/c mice (n = 48) were sensitized similarly as described above. Mice were then topically challenged 2 week post-sensitization on the dorsal side of a tape-stripped ear with 5 μl of antigen solution (2% BLG, 2% OVA or 0.2% PNA) in PBS. The allergen challenge was performed on the left ear while PBS was applied to the right ear. Mice receiving BLG, OVA or PNA during sensitization and challenge, as described above, were termed as 'BLG SC', 'OVA SC' or 'PNA SC', respectively. Another group of mice that received PBS during sensitization and challenge (PBS SC) was considered the negative control. A third group of mice that received PBS during sensitization and topical antigens during challenge was considered the challenge control group (BLG C, OVA C, PNA C or PBS C). Ear thickness was measured using a Quickmike digital micrometer (Mitutoyo, Japan) at 0, 0.5, 1, 2, 6 and 24 h post-challenge. The ear swelling response was determined as the percent difference between test (antigen challenged) and control (PBS challenged) ears [61].

### Measurement of serum total IgG1 and IgE levels by ELISA

Mice were sensitized and challenged as described above and then euthanized 24 h post challenge using CO_2_. Blood was collected by cardiac puncture and centrifuged at 20°C for 15 min at 700 × g to obtain serum. Serum samples were stored at -70°C. Mouse IgG1 and IgE ELISAs (Bethyl Laboratories Inc., Montgomery, TX) were performed according to manufacturer's instructions with minor modifications. Serum samples were diluted 1:100,000 for IgG1 and 1:20 for IgE ELISA assays. Supplied goat anti-mouse IgG1 (1:150,000) or IgE (1:20000) HRP conjugates were diluted accordingly. The plates were washed with the supplied wash buffer using a MW 96 microplate washer (Beckman Coulter, CA, USA). The absorbance was read at 450 nm using a Victor 1420 Multilabel Counter machine (Perkin Elmer, MA, USA). The concentrations of IgG1 and IgE immunoglobulins in the samples were calculated by comparison with a standard curve according to the manufacturer's instructions. All steps were performed at 20°C, unless otherwise mentioned. IgG1 and IgE ELISA kits have a detection limit of 250-3.9 ng/ml with inter- and intra-assay coefficients of variation (CV) of 10%.

### Measurement of plasma histamine levels

A third group of BALB/c mice (n = 24) were sensitized as described above and challenged i.p. 2 week post-sensitization with 50 μl of the same antigen solution. These mice were then euthanized 20 min post-challenge and heparinized blood collected and centrifuged for 15 min at 20°C at 700 × g to obtain plasma. Samples were frozen at -70°C until analyzed. Plasma histamine concentrations were measured using the ELISA kit manufactured by Labor Diagnostika Nord GmbH & Co. (MEDICORP Inc., Montreal, QC) and the concentration of histamine in the samples was calculated by comparison with a standard curve according to the manufacturer's instructions. This ELISA kit has a detection limit of 0.1-30 ng/ml with inter- and intra-assay CV of 12%.

### Passive Cutaneous Anaphylaxis (PCA) assay

The PCA assay was conducted according to Dearman and Kimber [20] and Li *et al. *[33] with minor modifications. The abdomen of each naïve mice was shaved and then intra-dermally (i.d.) injected with 30 μl of undiluted serum from an individual mouse that had been previously sensitized and challenged with the antigens as described above. Mice in the control group received the same amount of undiluted serum from PBS SC or naïve mice. After 48 h of serum sensitization, each mouse received an intra-venous (i.v.) challenge of 100 μl of the relevant antigenic solution (2.5 μg/μl) combined with 0.5% Evans blue dye in PBS. The appearance of a blue wheal at the site of i.d. serum injection was observed within 30 min following antigen challenge, and a reaction diameter of 3 mm or more was considered a positive PCA response.

### Statistical analysis

Data from the ear swelling response, and IgG1 and IgE ELISA experiments were statistically analyzed using SAS software (SAS Institute Inc., NC, USA). The data were log transformed prior to analysis in order to stabilize the variance. These experiments were designed as a randomized complete block design. Experiments involving the ear swelling response, and serum IgG1 and IgE ELISA consisted of 4 blocks; 2 groups (SC and C) and 4 treatments (BLG, OVA, PNA and PBS) in a factorial arrangement.

The mixed model method was used to analyze the repeated ear swelling measurements over time according to the method provided by Wang and Goonewardene [62]. The best fitting variance structure over time for antigen treatment and percent ear swelling was determined according to the Akaike criterion [62]. Linear and quadratic orthogonal polynomial contrasts over time and interactions of these with antigen treatments and the PBS control were performed to compare the time trends of ear swelling among the groups.

When analyzing the IgG1 and IgE ELISA data, Brown and Forsythe's [63] test was applied initially to compare the variances among groups using a general linear model (GLM) method. Mixed model methods were used for the analysis, with separate variances fit for each of the 2 groups (SC and C) when necessary. Multiple comparisons among the treatments within groups were performed and Bonferroni's correction was used to adjust the p-values to control for the type-1 error rate. For the histamine ELISA data, a mixed model procedure was used to determine the least squares means (LSM) and a Dunnett's test was performed to compare the LSM of the allergen treated groups to the PBS control group. A Student's t-test was performed to determine the statistical significance of the PCA data. P-values less than or equal to 0.05 (p ≤ 0.05) were considered to be statistically significant.

## List of abbreviations

MLN: Mesenteric Lymph Node; i.p.: intraperitoneal; BLG: β (beta)-lactoglobulin; OVA: ovalbumin; PNA: peanut agglutinin; h: hour; min: minutes; cDNA: complementary DNA; GO: gene ontology; IgG1: immunoglobulin G1; IgE: immunoglobulin E; LSM: least squares means; PBS: phosphate buffered saline; PCA: Passive Cutaneous Anaphylaxis; RT-PCR: reverse transcription-polymerase chain reaction; SC: sensitization and challenge; C: challenge; SE: standard error; IL-13: interleukin 13; IL-4: interleukin 4; IL-2: interleukin 2; IFN-γ: interferon gamma; ELISA: enzyme-linked immunosorbent assay; Th1 cells: type-1 T helper cells; Th2 cells: type-2 T helper cells; TNF-α or β: tumor necrosis factor alpha or beta; TMB: 3,3',5,5'-tetramethylbenzidine; CCAC: Canadian Council on Animal Care; i.v.: intra-venous; GLM: general linear model.

## Authors' contributions

MH, HB and NK participated in designing and planning of the experiments. MH and NK carried out the animal experiments. MH conducted all of the immunological and molecular genetic studies, and drafted the manuscript. HB and NK revisited the manuscript critically for important intellectual content. MH, HB and NK have read and approved the final manuscript.

## Acknowledgements

This research was supported by the Ontario Ministry of Agriculture, Food and Rural Affairs (OMAFRA), and University of Guelph. Authors gratefully thank Dr. Margaret Quinton for assisting with the statistical analysis and Ms. Quimei You for her assistance with the animal experiments. Authors would also like to thank Ms. Jing Zhang at the University of Guelph Genomics Facility for her support with the equipments.

## Supplementary Material

Additional file 1 **Genes significantly differentially expressed in response to BLG, OVA and PNA sensitizations (p < 0.05)**. Gene expression data Click here for file

Additional file 2 **Summary of overlapping genes commonly expressed in response to BLG, OVA and PNA sensitization with their corresponding fold changes and p-values**. Gene expression data Click here for file

Additional file 3 **List of significant GO biological processes enriched (p ≤ 0.05) with the genes significantly differentially expressed (p ≤ 0.05) in response to BLG sensitization**. Gene ontology data Click here for file

Additional file 4 **List of significant GO biological processes enriched (p ≤ 0.05) with the genes significantly differentially expressed (p ≤ 0.05) in response to OVA sensitization**. Gene ontology data Click here for file

Additional file 5 **List of significant GO biological processes enriched (p ≤ 0.05) with the genes significantly differentially expressed (p ≤ 0.05) in response to PNA sensitization**. Gene ontology data Click here for file

Additional file 6 **Common GO biological processes enriched with the genes differentially expressed in response to BLG, OVA and PNA sensitizations**. Gene ontology data Click here for file

Additional file 7 Effects of BLG, OVA or PNA sensitization and challenge (SC) on the ear swelling of BALB/c mice. Overall ear swelling responses observed in BLG, OVA and PNA SC groups of mice were not significantly different from each other. Results expressed as LSM ± SE. * p > 0.3; *** p < 0.001. Color figure (bar graph) Click here for file
