# Supplementary figures and images for: Mesenteric lymph node transcriptome profiles in BALB/c mice sensitized to three common food allergens
Source: BMC Genomics. 2011 Jan 6;12:12. doi: 10.1186/1471-2164-12-12 (PMC3023748; doi:10.1186/1471-2164-12-12)

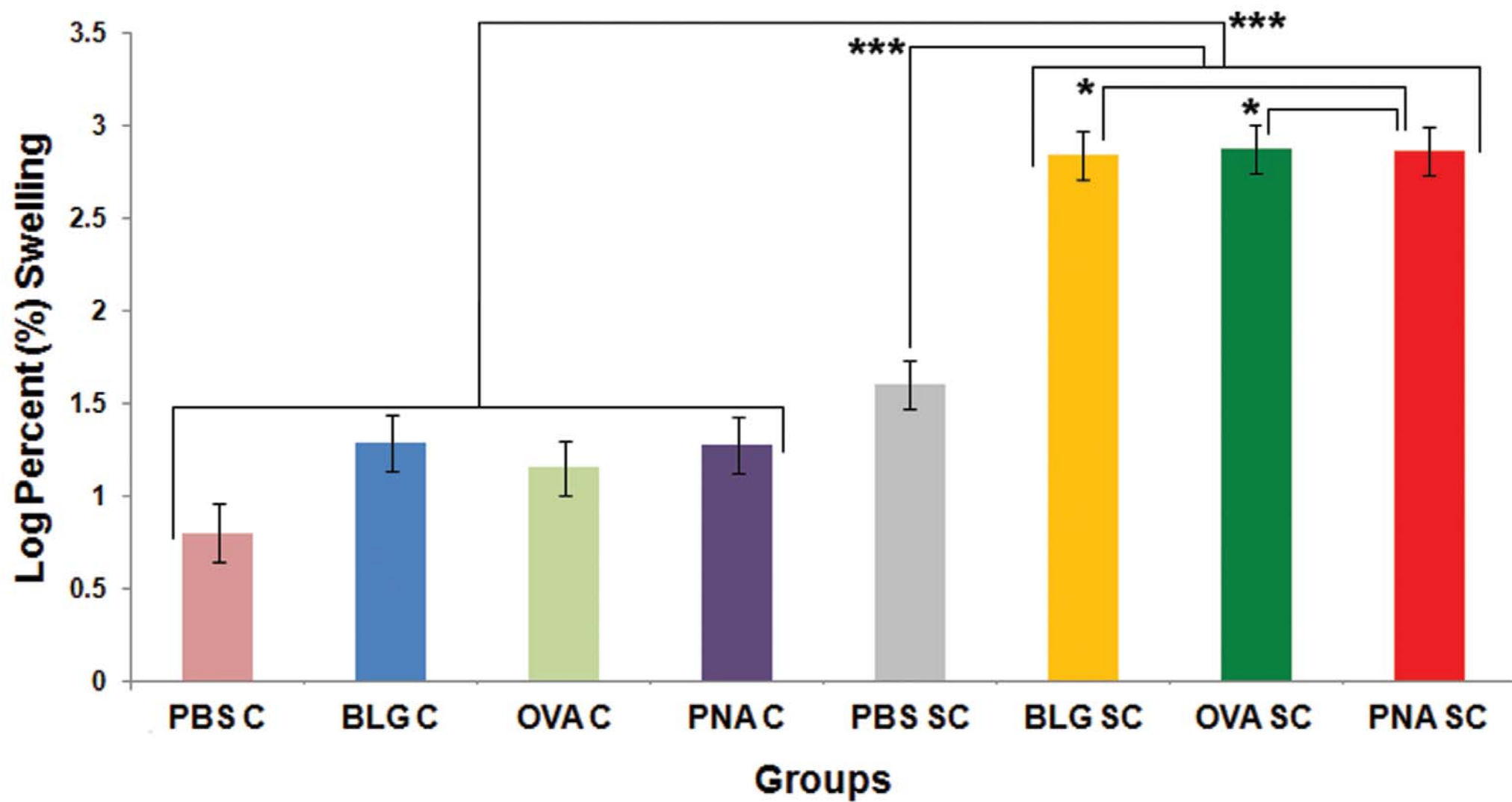

Supplement: Additional file 7 — Effects of BLG, OVA or PNA sensitization and challenge (SC) on the ear swelling of BALB/c mice. Overall ear swelling responses observed in BLG, OVA and PNA SC groups of mice were not significantly different from each other. Results expressed as LSM ± SE. * p > 0.3; *** p < 0.001. Color figure (bar graph) [file 1471-2164-12-12-S7.PDF]
